# Supplementary figures and images for: Covichem: A biochemical severity risk score of COVID-19 upon hospital admission
Source: PLoS One. 2021 May 6;16(5):e0250956. doi: 10.1371/journal.pone.0250956 (PMC8101934; doi:10.1371/journal.pone.0250956)

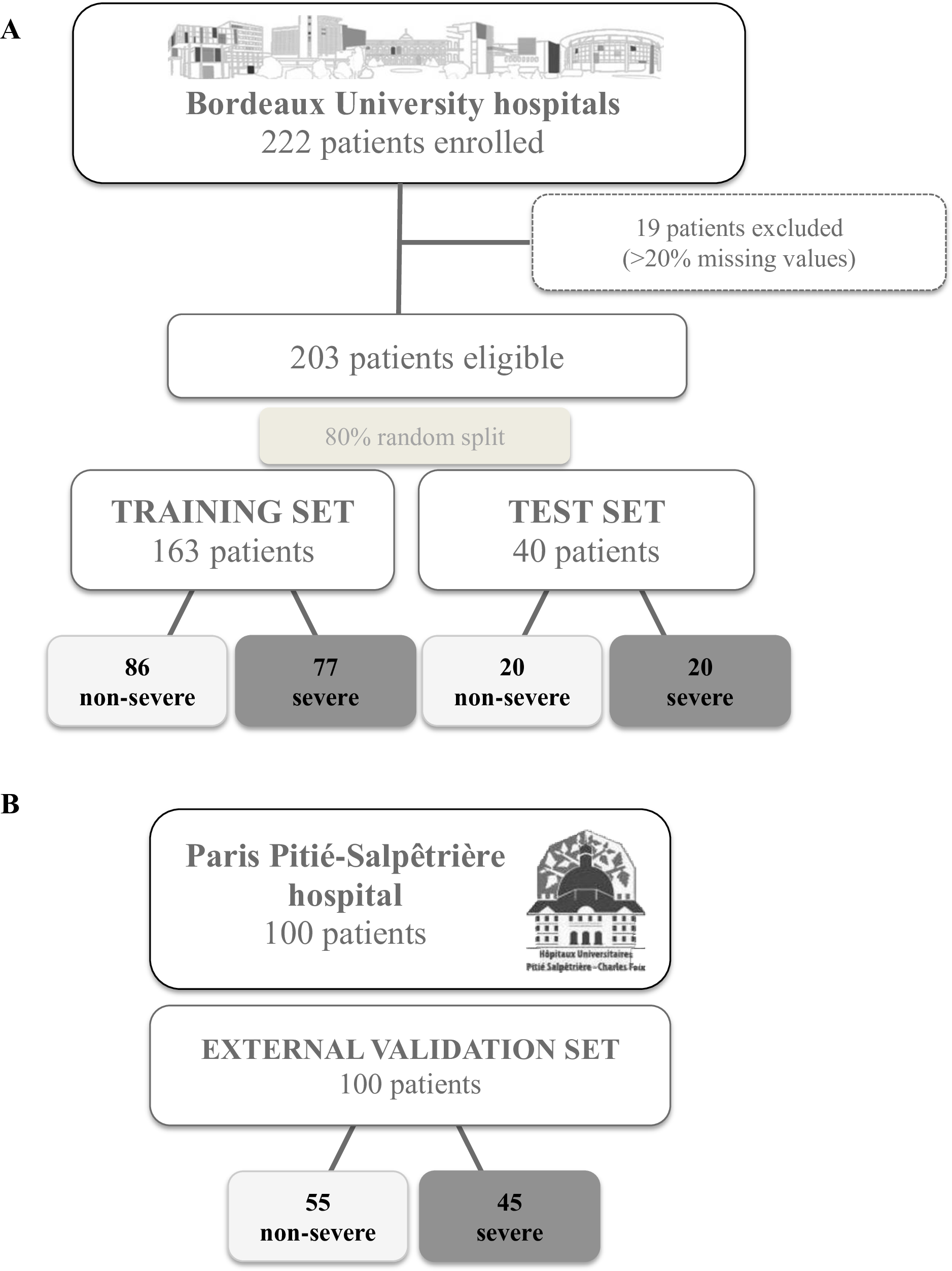

Supplement: S1 Fig — (TIF) [file pone.0250956.s001.tif]

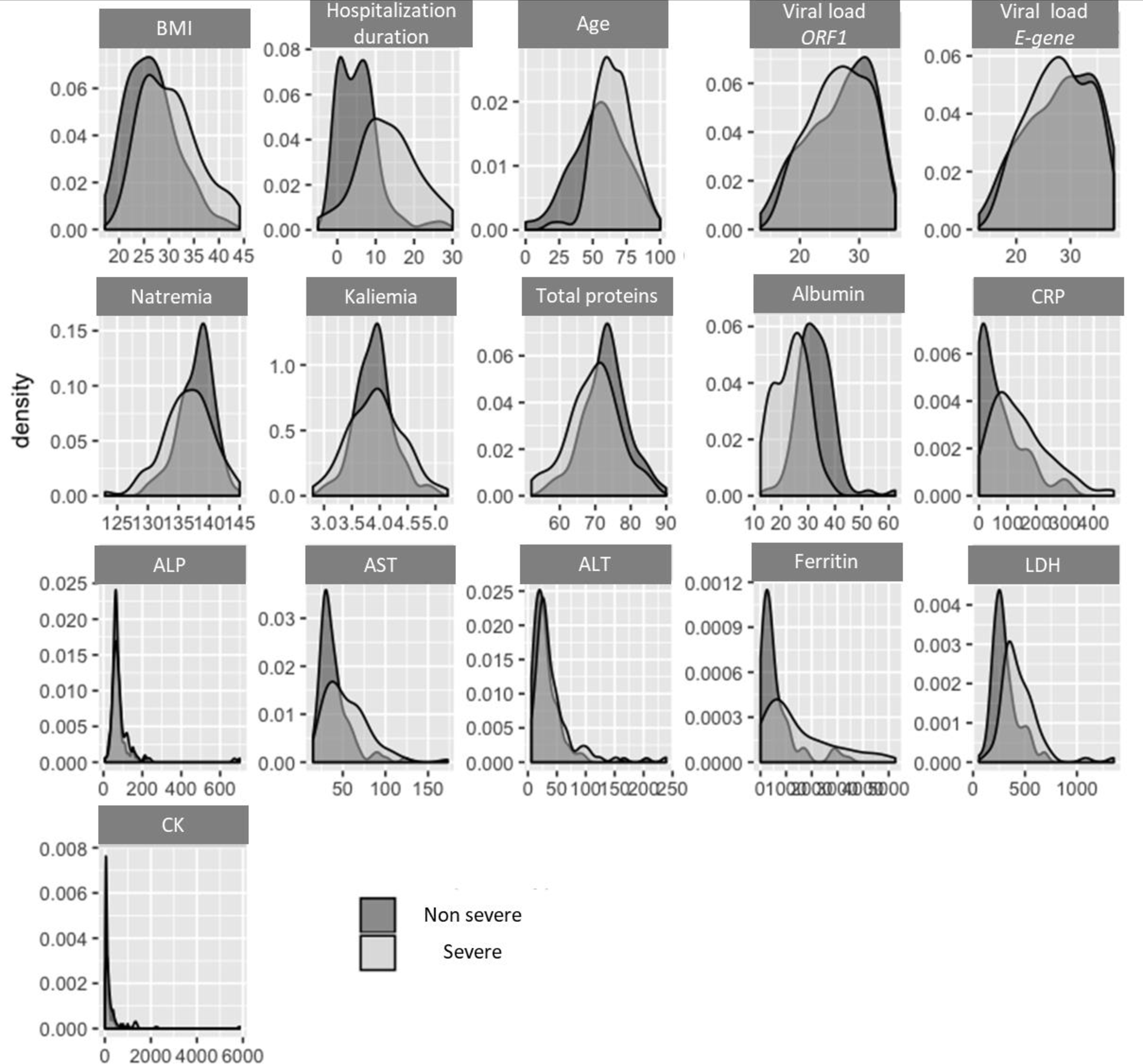

Supplement: S2 Fig — ALP, Alkaline Phosphatase, AST, Aspartate Aminotransferase; ALT, Alanine Aminotransferase; BMI, Body Mass Index; CK; Creatine Kinase; CRP, C-reactive protein; LDH, Lactate Dehydrogenase. (TIF) [file pone.0250956.s002.tif]

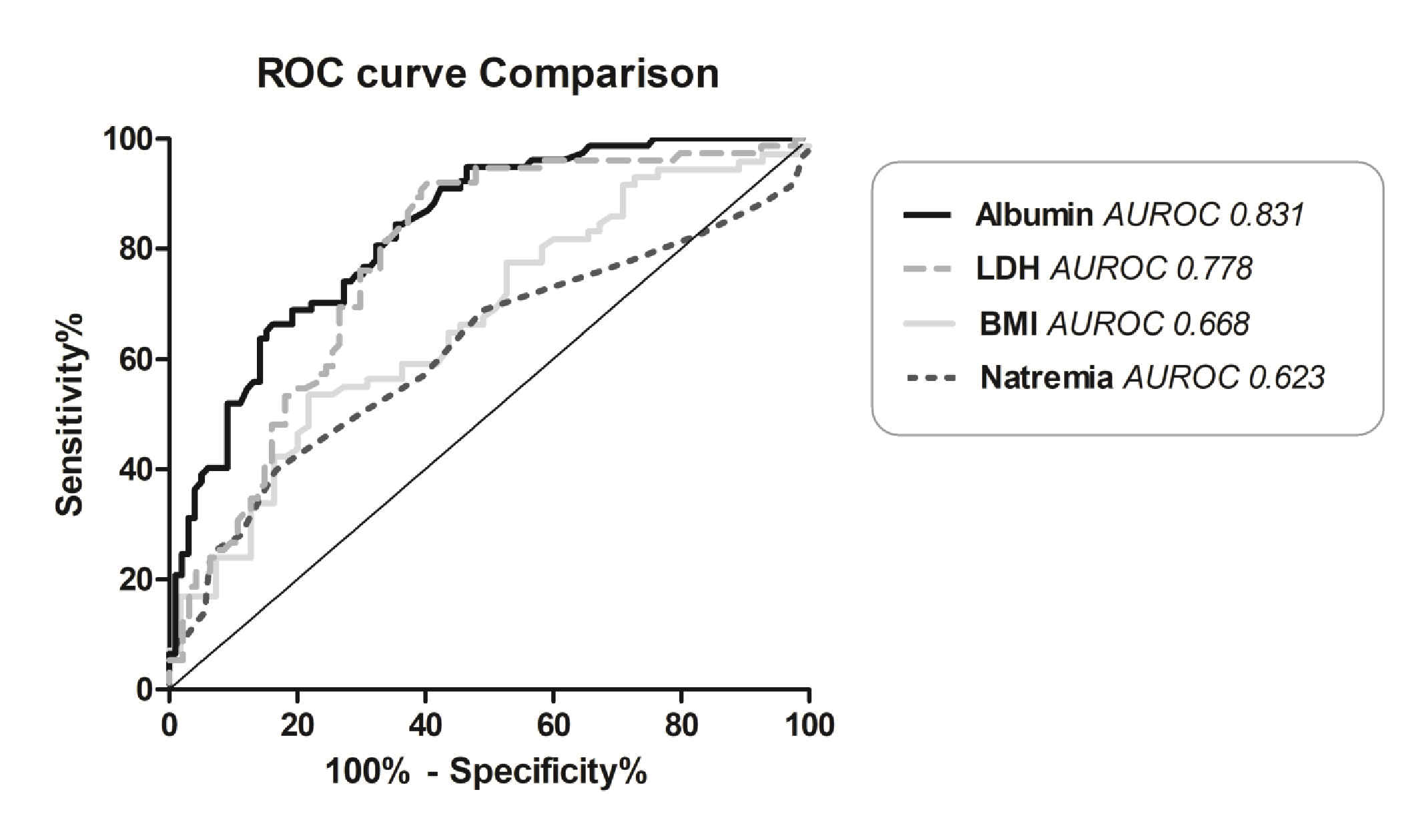

Supplement: S3 Fig — The areas under Receiver Operating Characteristic curves (AUROC) are indicated for each variable on the graph legend. BMI, Body Mass Index; LDH, Lactate Dehydrogenase. (TIF) [file pone.0250956.s003.tif]

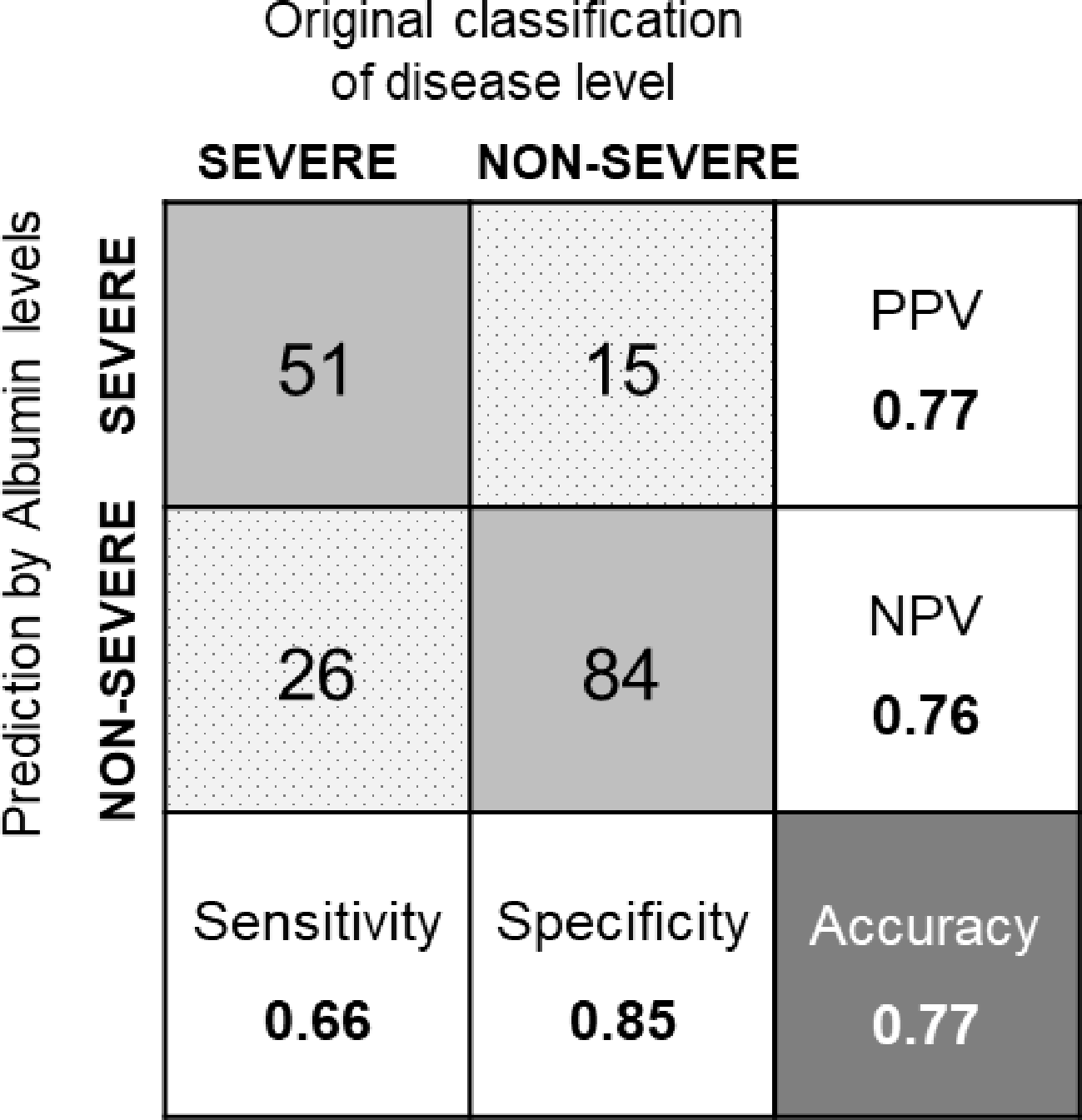

Supplement: S4 Fig — Grey squares correspond to true positive and true negative values, spotted grey squares represent false positive and false negative values. Predictions were calculated for a cut-off of albumin at 26.95 g/L. NPV, Negative Predictive Value; PPV, Positive Predictive Value. (TIF) [file pone.0250956.s004.tif]
