# Supplementary material for: Covichem: A biochemical severity risk score of COVID-19 upon hospital admission
Source: PLoS One. 2021 May 6;16(5):e0250956. doi: 10.1371/journal.pone.0250956 (PMC8101934; doi:10.1371/journal.pone.0250956)
Supplement: S1 Table — ALP, Alkaline Phosphatase; ALT, Alanine Aminotransferase; AST, Aspartate Aminotransferase; BMI, Body Mass Index; CK, Creatine Kinase; CRP, C-reactive protein; LDH, Lactate Dehydrogenase. (PDF) [file pone.0250956.s005.pdf]

Variable	Pearson correlation coefficients
Albumin	0.55
Hospitalization duration	0.48
LDH	0.40
Ferritin	0.38
CRP	0.34
AST	0.31
BMI	0.29
Obesity	0.28
Cardiovascular disease	0.26
Age	0.24
Hypertension	0.23
CK	0.21
Total proteins	0.19
Natremia	0.18
Sex	0.18
ALP	0.13
ALT	0.13
Diabetes	0.12
Smoking	0.09
Infectious disease	0.09
Viral load E gene	0.07
Renal disease	0.06
Dyslipidemia	0.05
Cancer	0.02
Kaliemia	0.02
Inflammatory disease	0.02
Viral load ORF1	0.02
Liver disease	0.01
Respiratory disease	0.01
