# Supplementary material for: Covichem: A biochemical severity risk score of COVID-19 upon hospital admission
Source: PLoS One. 2021 May 6;16(5):e0250956. doi: 10.1371/journal.pone.0250956 (PMC8101934; doi:10.1371/journal.pone.0250956)
Supplement: S2 Table — Continuous variables are expressed as median (25th, 75th percentile). Discrete variables are presented as absolute (relative) frequencies of patients. ALP, Alkaline Phosphatase; ALT, Alanine Aminotransferase; BMI, Body Mass Index; CK, Creatine Kinase; CRP, C-reactive protein; AST, Aspartate Aminotransferase; Ct, Cycle threshold; LDH, Lactate Dehydrogenase; NA, not available. (PDF) [file pone.0250956.s006.pdf]

Variable	Training set (n=163)		Test set (n=40)		External Validation set (n=100)	
	**Non-severe (n=86)**	**Severe (n=77)**	**Non-severe (n=20)**	**Severe (n=20)**	**Non-severe (n=55)**	**Severe (n=45)**
**Age (years)**	59.5 (48, 71)	66 (57, 76)	56 (49 ,85.3)	72 (62, 77)	58 (44,73)	57 (53, 64)
**Male sex, n (%)**	51 (59%)	28 (36%)	9 (45%)	8 (40%)	25 (45%)	34 (75%)
**Diabetes, n (%)**	13 (15%)	17 (22%)	3 (15%)	6 (30%)	NA	NA
**Dyslipidemia, n (%)**	17 (20%)	17 (22%)	5 (25%)	6 (30%)	NA	NA
**Hypertension, n (%)**	25 (29%)	38 (49%)	7 (35%)	11(55%)	NA	NA
**Smoking, n (%)**	15 (17%)	21 (27%)	2 (10%)	1 (5%)	NA	NA
**Obesity, n (%)**	11 (13%)	26 (34%)	2 (10%)	8 (40%)	5 (9%)	12 (27%)
**BMI (kg/m²)**	25.6 (24.3, 27.1)	27.3 (25.6, 31.7)	26.2 (25.5, 26.6)	28.1 (25.6, 33.3)	NA	NA
**Cardiovascular disease, n (%)**	17 (20%)	36 (46%)	6 (30%)	7 (35%)	5 (9%)	26 (58%)
**Cancer, n (%)**	14 (16%)	16 (20%)	4 (20%)	1 (5%)	NA	NA
**Infectious disease, n (%)**	2 (2%)	2 (2.5%)	4 (20%)	0	NA	NA
**Inflammatory disease, n (%)**	11 (13%)	9 (12%)	2 (10%)	1 (5%)	NA	NA
**Liver disease, n (%)**	2 (2%)	1 (1%)	1 (5%)	2 (10%)	NA	NA
**Renal disease, n (%)**	2 (2%)	6 (8%)	2 (10%)	0	NA	NA
**Respiratory disease, n (%)**	20 (23%)	18 (23%)	4 (20%)	2 (10%)	NA	NA
***ORF1* (Ct value)**	28.8 (23.5, 31.1)	29 (24.2, 31.1)	27.6 (23.7, 30.3)	28.2 (26, 29.5)	NA	NA
***E-gene* (Ct value)**	29.7 (24.2, 34)	29.9 (25.2, 32.6)	28.4 (24, 32.1)	29.3 (26.7, 30.2)	NA	NA
**Natremia, mmol/L**	139 (136, 140)	137 (135,139)	138 (137, 139)	136(133, 138)	138 (137,140)	138 (135,142)
**Kaliemia, mmol/L**	3.92 (3.67, 4.10)	3.91 (3.63, 4.22)	3.88 (3.74, 3.99)	3.99 (3.51, 4.15)	NA	NA
**Total proteins, g/L**	73 (68, 76)	71 (66, 75)	75 (71, 79)	71 (67, 75)	NA	NA
**Albumin, g/L**	31.9 (27.8, 36.3)	24.3 (20, 28.3)	33.2 (30.5, 35.7)	23.7 (20.5, 25.8)	32 (30, 35)	25 (22, 29)
**CRP, mg/L**	56.9 (10.9, 129)	135 (68, 192)	60 (20, 84)	110 (69, 233)	NA	NA

**ALP, U/L**	67 (59, 84)	68 (59, 89)	65 (54, 70)	79 (63, 106)	NA	NA
**AST, U/L**	37 (30, 46)	50 (35, 68)	37 (27, 46)	49 (41, 70)	NA	NA
**ALT, U/L**	26 (18, 42)	33 (24, 48)	27 (19, 43)	37 (27, 46)	NA	NA
**Ferritin, ng/mL**	437 (195, 818)	1056 (601, 2119)	352 (248, 646)	920 (387, 1266)	558 (205, 865)	1785 (1108, 3280)
**LDH, U/L**	295 (237, 367)	400 (340, 483)	272 (243, 303)	433 (365, 492)	322 (246, 431)	478 (418, 595)
**CK, U/L**	75 (37, 161)	140 (62, 270)	111 (68, 179)	213 (98, 798)	75 (32, 105)	250 (133, 466)
