# Supplementary material for: Covichem: A biochemical severity risk score of COVID-19 upon hospital admission
Source: PLoS One. 2021 May 6;16(5):e0250956. doi: 10.1371/journal.pone.0250956 (PMC8101934; doi:10.1371/journal.pone.0250956)
Supplement: Attachment — Submitted filename: Point by point reply-PlosOne.docx [file pone.0250956.s008.docx]

**Point by point reply:**

We would like to thank the reviewer and the editor for their fair review of our work.

*Line 52 -55: It is not clear what “soon after the first phase of COVID-19” refer to, is this first phase in France? what timeline?*

We now specify the timeline as being April 2020.

Additionally, “few” parameters seem inaccurate when taking into account that Rod et al. 2020 reported 60 risk factors for disease severity using early studies coming from China and Singapore with at least 50% of those been biomarkers that could be tested in blood.

We agree that several (even now numerous) studies have described risk factors for COVID-19 severity. However, when undertaking the present study, our analysis of the literature, focused to biochemical parameters, showed that only a few were consistently found linked to disease severity and not to other outcomes (such as death ratio, or hospitalization rates). We thank the reviewer for providing the “Rod, J. E., Oviedo-Trespalacios, O., & Cortes-Ramirez, J. (2020). A brief-review of the risk factors for covid-19 severity. Rev Saude Publica, 54, 60. doi:10.11606/s1518-8787.2020054002481” reference, that we now cite, and which summarizes the available data on COVID severity in April 2020. As shown in the Table of this review, CRP, D-Dimer and Albumin show consistency between studies. We changed the introduction of the manuscript accordingly, and cite Rod et al.

*Line 68 – 69: The following sentence was not fully clear to me “Turn-around-time is short to obtain automated biochemical parameters 70 measures (<2h)”. I would suggest to double check English and the connectors between sentences in the last paragraph of the introduction. The authors seem to follow the convention of writing the aim of the research at the end of the introduction. However, the last sentence sound more like an action/task that belongs to the methods section and it does not appear to me to be written as an aim.*

We agree with the reviewer’s comment, and we now limited the last sentence of the introduction to the aim of the study, which was to identify biochemical parameters useful to establish a COVID-19 severity score.

*Line 122-123: It is not fully clear what “referring” to odds ratios means. Additionally, odds ratios are a different mathematical construct than probability ratios (which are commonly called risk ratios under an epidemiological and the quantitative notions of risk). Generally in epidemiology both odds ratios and risk ratios are called measures of association between variables, it might be worthwhile to consider the use the term “measures of association”. This would likely make a value free statement in the results section and leave value judgements about risk for the discussion and the conclusion.*

*Table 1: There is a risk that the use of odds ratios overestimates relative risks when the rare disease assumption (prevalence less than 10%) is not present. This seems to happen for most comorbidities in Table 1. This could mean that the presented odds estimates might be overestimated. If the interest is risk, why use logistic regression instead of other generalized linear models (e.g. log binomial model) that offer relative risk as the output and not odds ratios?.*

The reviewer is right to underline that according to the mathematical calculation of odd ratios, there is a risk of overestimation of the studied effects. We originally provided these data as supplementary data, because odd ratios are commonly provided in such studies. However, we did not determine the score based on these results and propose to withdraw supplemental Table S2 as the part in text referring to this Table and odd ratios.

*Line 300 – What machine learning model? I could not find any machine learning model in the methods section.*

It is specified in the M&M and in the result sections: we used a stepwise model selection by Akaike Information Criterion.

*Should there be a table comparing existing predictors against the proposed model?*

We did compare the best single predictors, found by others over the literature, with the proposed score by building ROC and calculating AUROC (Figure S3). There are no published ‘”existing single predictors”. In addition, as mentioned in the discussion we compared our score to the “CALL” score.

*I think the discussion should be supplemented with discussions of other papers that are addressing the topic. Eg.*

All the mentioned studies are now cited in the discussion.

*There should be a discussion regarding the external validity of the score beyond the used sample sizes in the particular populations that were used to propose and validate. Why should other clinicians in Europe/ developed nations expect similar results?. What about developing nations?.*

We provide this in the conclusion.

*Lines 359 – 361: Rod et al. 2020 review of early studies coming from china looking at risk factors for a composite index of severe-fatal the COVID-19 disease propose albumin as one of the most consistent risk factors for severe-fatal COVID-19. Based on this information, it is hard to understand how the present paper is “repositioning” albumin as a standard of care of any COVID-19 disease patient admitted to urgent care. Please be more specific what is the contribution that the present paper is adding to the literature in regards albumin levels. The conclusion needs a lot of work. I found hard to find the logic between the aim being to propose, validate and promote the use of a score by using an app and to conclude that the paper has reposition albumin as an important risk factor (which is not accurate based on the above-mentioned rationale). What future research directions does the current state of development of this score lead researchers to?*

We agree with the reviewer that mentioning albumin at this stage of the manuscript was not appropriate. The sentence regarding albumin is now removed. We just kept the discussion part about albumin in the discussion section.

We thank the reviewer for suggesting to open the study to future directions. We now provide a conclusion in the context of the disease evolution with the new variants.
